# Application of *Chlorella vulgaris* Beijerinck as a Biostimulant for Growing Cucumber Seedlings in Hydroponics

**DOI:** 10.3390/biotech12020042

**Published:** 2023-05-22

**Authors:** Galiya I. Vildanova, Rezeda Z. Allaguvatova, Dina F. Kunsbaeva, Natalia V. Sukhanova, Lira A. Gaysina

**Affiliations:** 1 Department of Bioecology and Biological Education, M. Akmullah Bashkir State Pedagogical University, Oktyabrskoy Revolyutsii Street 3-a, 450008 Ufa, Russia; ilshatovna2594@mail.ru (G.I.V.); kunsbaevad@yandex.ru (D.F.K.); n_suhanova@mail.ru (N.V.S.); 2 Laboratory of Botany, Federal Scientific Center of the East Asia Terrestrial Biodiversity, Pr-t 100-let Vladivostoka, 159, 690022 Vladivostok, Russia; allaguvatova@yandex.ru; 3 All-Russian Research Institute of Phytopathology, Institute Street, 5, 143050 Bolshye Vyazemy, Russia

**Keywords:** algae, authentic strain, dry biomass, growth stimulator, roots, shoots, seedlings, simple hydroponic system, stimulating effect, plants

## Abstract

Hydroponics is a promising method for growing agricultural plants and is especially relevant in the context of global climate change. Microscopic algae, including *Chlorella vulgaris*, has great potential for use in hydroponic systems as natural growth stimulators. The effect of the suspension of an authentic strain of *Chlorella vulgaris* Beijerinck on the length of cucumber shoots and roots, as well as its dry biomass, was studied. During cultivation in a Knop medium with the addition of *Chlorella* suspension, the length of the shoots was shortened from 11.30 to 8.15 cm, while the length of the roots also decreased from 16.41 to 10.59 cm. At the same time, the biomass of the roots increased from 0.04 to 0.05 g. The data obtained indicate the positive effect of the suspension of the *Chlorella vulgaris* authentic strain on the dry biomass of cucumber plants in hydroponic conditions and make it possible to recommend this strain for use when growing plants in hydroponic systems.

## 1. Introduction

World population growth, a decrease in cultivation areas, water deficiency, and global warming are serious problems for food production around the world [1]. An important difficulty is the reduction in suitable agricultural territories. Soil is the most appropriate substrate for these plants [1,2]. It provides plants with nutrients, water, and minerals [3,4]. In many regions, there has been a reduction in fertile agricultural lands due to their unfavorable geographical or topographic conditions [5,6]. Moreover, land degradation, low humus content, acidic or alkaline soil pH, poor drainage, and soil contamination by pathogens and toxicants prevent plant growth [7]. The crop yield depends on weather conditions, and a significant part of the crop can be lost due to droughts and floods [8,9].

One of the ways to grow plants, regardless of extreme environmental conditions, is the use of hydroponics. Hydroponics is a unique method for growing plants using an aqueous solution as a source of nutrients [4]. The definition of hydroponics has been broadened over the years, and it is now almost a synonym for soilless culture [10]. This refers to any solid or liquid inert substrate plant cultivation [11]. Gravel, sand, vermiculite, perlite, expanded clay, and rockwool are solid substrates for hydroponics. In fact, most plants cultivated in greenhouses, container nurseries, balconies, flats, and homes grow hydroponically [10].

The role of algae in hydroponics has been discussed for a long time. Both the positive and negative effects of algae on plants in hydroponic systems have been analyzed. There is information about the stimulating effect of the brown algae *Ascophyllum nodosum* and vermicompost extracts on the number of leaves and plant height, stem diameter, wet and dry weight of the stem, and quality traits of the cherry tomato [12]. Algal photosynthesis in culture media can supply O_2_, which is important for root respiration and growth [13,14]. Algae secrete growth stimulants, such as auxins, cytokinins, gibberellins, ethylene, and abscisic acid [15,16,17,18,19,20,21,22]. Algae has a stimulating influence on the balancing of the pH drop caused by nitrifying bacteria in the floating-raft aquaponic system. These organisms are more productive for nitrogen elimination than vegetables [23]. The negative influence of algae is caused by competition with high plants for nutrients, resulting in the clogging and growth of organic carbon content [14,24,25]. Moreover, algae could suppress plant growth due to the production of toxins [26].

Algae of the genus *Chlorella* are among the most commonly used algae in biotechnology [27,28,29,30,31,32,33,34,35,36,37]. *Chlorella* is very popular due to its rapid growth and ability to survive in a wide range of environmental conditions, including extreme levels of temperature, pH, salinity, and other factors [38].

There are some data regarding the use of *Chlorella* in hydroponics. In a previous study, hydroponic wastewater as a potential culture medium for *Chlorella vulgaris* growth was established, indicating that this alga efficiently eliminated nitrogen and phosphorus from hydroponic wastewater. This is important for recycling wastewater [39]. *Chlorella vulgaris* was effective in bioremediating hydroponic wastewater and producing biomass in different cultivation conditions [40]. During the co-cultivation of Swiss chard and *Chlorella vulgaris,* a high number of leaves (18.56%), total fresh weight (17.13%), and root volume (36.98%) in comparison with Swiss chard in Hoagland’s growth medium alone were observed [41]. When growing spinach in a floating culture with the addition of *Chlorella vulgaris* together with a mix of beneficial bacteria and mycorrhiza plant quality parameters, the total phenols, vitamin C, total soluble solids, chlorophyll, titratable acidity, iron, phosphorus, potassium, magnesium, manganese, and zinc concentrations of the leaves increased. The nitrate concentration in the young spinach leaf was substantially reduced [42]. A study of different *Chlorella* strains in hydroponic horticulture of lettuce, pakchoi, rocket, spinach, and basil in residual waters of *Oreochromis niloticus* aquaculture with biofloc technology in different cultivation conditions revealed that wastewater with the addition of *Chlorella* sp. was the most favorable for growing plants [43]. Using *Chlorella vulgaris* for hydroponically grown lettuce made it possible to reduce mineral fertilizers up by to 60% [44].

However, despite the available publications, the biotechnological potential of *Chlorella vulgaris* in hydroponics has not yet been fully studied. This is especially true for a vegetable crop as valuable as a cucumber (*Cucumis sativus* L.). The cucumber plant belongs to the family Cucurbitaceae [45]. It is the most grown plant of this family. Cucumber belongs to the annual trailing plants. It has underground roots and an aboveground stem that grows on support. Cucumber has large-sized leaves, forming a canopy-like structure above its fruit. The cucumber is characterized as a fruit due to its dicotyledonous and covered seeds that emerge from the flowers [46].

The cucumber is a very old, cultivated plant that is grown in almost all countries of temperate zones [47]. Cucumbers contain many important nutrients, and they are a low-calorie valuable product [45]. It has been demonstrated to have various medicinal properties, including antimicrobial and antioxidant activities, as well as a glycemic-lowering ability. The antioxidant, anticholinesterase, and antimonoamine oxidase properties of cucumber and cabbage extracts have been reported. Moreover, cucumber extract inhibits lipid peroxidation in the human brain. Cucumber demonstrates enzyme-inhibiting properties, which are connected with neurodegenerative diseases. Cucumber extract contains phenolic compounds such as quercetin and gallic and caffeic acids. This plant produces a high range of different compounds that can protect against cancer and cardiovascular disease. These compounds also have anticancer activity. Various biologically active compounds, also called phytochemicals, are detected in cucumber. These belong to the alkaloids, flavonoids, steroids, saponins, tannins, and phlobatannins [48]. Cucumber is a very popular vegetable. The most popular varieties of cucumber originate from Europe, America, China, the Himalayan Mountains, and India [46]. In Asia, cucumber is the fourth most extensively grown vegetable after tomatoes, cabbage, and onion, and in Western Europe, it is the second crop after tomato [45,49]. It is necessary to note that the yield of cucumber from hydroponics is higher compared to that from cultivation in the soil (4727.38 g/plant and 4427.38 g/plant, respectively) [50].

The aim of this study was to investigate the influence of suspension of a *Chlorella vulgaris* authentic strain on the length and biomass of the shoots and roots of cucumbers in hydroponic conditions.

## 2. Materials and Methods

### 2.1. Description of Chlorella vulgaris Suspension and Strain Used in This Study

**Figure 1:**
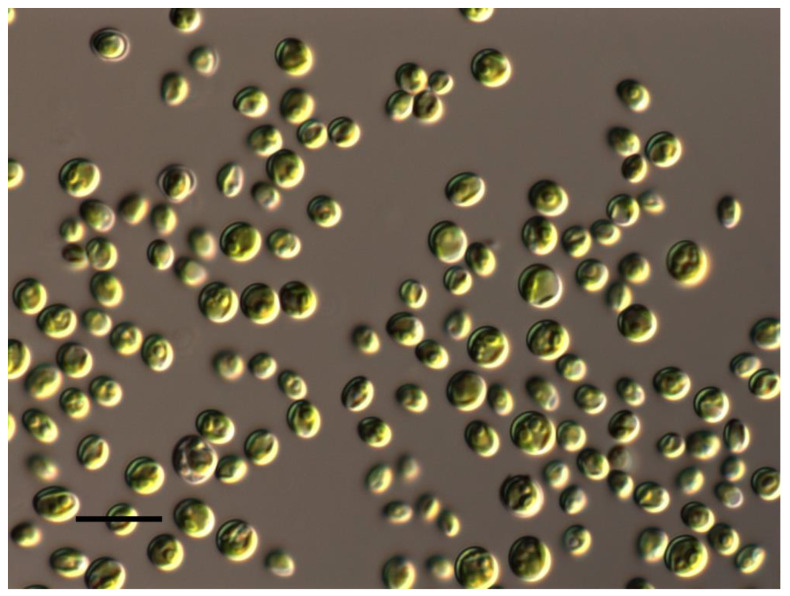
Morphology of the authentic strain of *Chlorella vulgaris.* Scale bar—10 µm.

*Chlorella vulgaris* suspension is a pale green liquid containing liquid media, alga cells, and products of its metabolism. An authentic (reference) strain of *Chlorella vulgaris* Beijerinck (SAG 211-11b, BCAC 76, CCAP 211/11B, UTEX 259) was used in this study. An authentic (reference) strain is the strain on the basis of which the species is described. It is a reference sample with which other strains can be compared to determine their belonging to the species. *Chlorella vulgaris* SAG 211-11b was isolated in the year 1989 from a pool near Delft in the Netherlands, and it is the type species of the genus *Chlorella* [51,52]. The algae have a very simple morphology. The cells are ellipsoid or spherical with a diameter of 2.3–5.3 µm, up to 5.5 µm during autospore formation. The chloroplast is wide-lobed or cup-shaped, with 2–4 starch grains. (Figure 1). It is necessary to note that when using an authentic chlorella strain, we did not need to conduct the genetic confirmation of the accuracy of the species definition.

### 2.2. Preparation of Chlorella vulgaris Suspension

*Chlorella* culture was maintained on a Bold liquid medium [53]. At 2 months before the start of the experiments; the algae were transferred into a Knop solution [54] with the following salt solution (per 1 L of water): Ca(NO_3_)_2_—1 g, KH_2_PO_4_—0.25 g, MgSO_4_—0.25 g; KCl—0.125 g, FeCl_3_—0.0125 g. The Knop solution is very popular when cultivating plants in hydroponic systems. *Chlorella vulgaris* suspension was cultured on a Knop solution at a temperature of 25 ± 5 °C and a 12:12 h light:dark cycle for two weeks.

For morphological observation of algae, an Axio Imager A2 (Carl Zeiss, Oberkochen, Germany) equipped with Nomarski DIC optics was used. *Chlorella* micrographs were made by an Axio Cam MRC (Carl Zeiss, Germany) camera at magnification ×1000 with oil immersion using AxioVision 4.9.1 software.

### 2.3. Preparation of Cucumber Seeds and Simple Hydroponic System

Cucumber seeds of the F1 “Crane” variety were used in the experiments. The F1 “Crane” variety is an average early one, and the period of time between the emergence of the seedlings and the collection of the first fruits is about 45 days. The growth of the main stem is significant and can reach a height of up to 190 cm. The plant itself is braided and capable of overgrowing with many lateral shoots.

Cucumber seeds were soaked in distilled water in Petri dishes for germination for 3 days. For this purpose, 2 layers of filter paper were placed on the bottom of the Petri dish, and the seeds were laid out. Then, water was poured into the dish until it was completely moistened and covered with another layer of filter paper. The Petri dishes were incubated at a temperature of 25 °C away from direct light sources.

**Figure 2:**
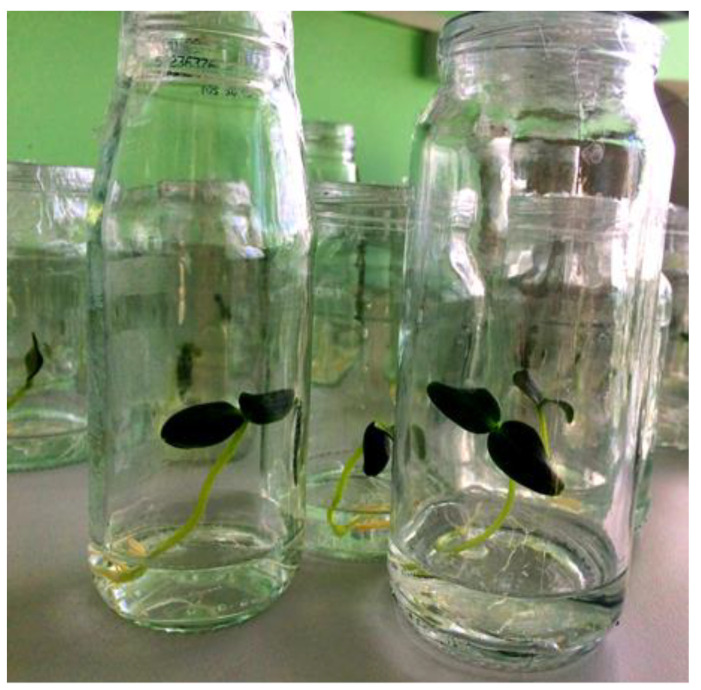
General view of the simple hydroponic system.

**Figure 3:**
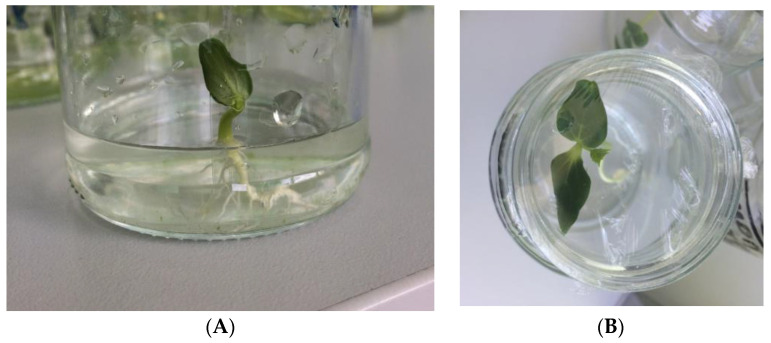
Peculiarities of the simple hydroponic system. (**A**) Cucumber seedling after 1 day of transfer to hydroponic system. The roots are covered by liquid; (**B**) Seedling after 4 days of cultivation in can, covered by polyethylene film.

Glass cans with a volume of 150–200 mL were used as a simple hydroponic system (Figure 2). Such systems have been used in previous studies [14]. In this system, microalgae and plants can effectively grow together in a glass container. A Knop solution was added to the *Chlorella vulgaris* suspension at a ratio of 1:1. The density of chlorella cells in the final suspension was 10^6^ algae cells per 1 mL. The density of the chlorella culture was determined using the Goryaev camera [55]. Then, the resulting suspension was poured into glass cans at a height of 3–4 cm so that it covered the root system of the cucumber plants (Figure 3A). The cans were covered with a polyethylene film, in which small holes were made to reduce evaporation (Figure 3B).

Germinated cucumber seeds were placed in cans with a suspension. The film was removed 6 days after the start of cultivation. The repeatability of the experiment was 100 (100 seeds and seedlings were tested in the experimental and control variants). Cucumber seedlings in the hydroponic system were cultivated for 14 days in natural light at a temperature of 20 °C. At the same time, the level of liquid in the cans was monitored. If necessary, it was topped up to the level of 3–4 cm.

### 2.4. Analysis of Experimental Results

The cucumber seedlings were taken out of cans and divided into shoots and roots. The length of the shoots and roots was measured using a ruler. To clarify the fine details of the morphology, a magnifying glass with 20-fold magnification was used. Parts of the seedlings were laid out on filter paper until completely dry. Then, the weight of each shoot and root was weighed on Ohaus Pioneer PA214C analytical scales.

During statistical analysis, the values of the arithmetic mean, its error, median, standard deviation, and coefficient of variation were calculated [56]. The reliability of the research results was determined using Student’s *t*-test [57]. The statistical analysis of the results was carried out using Statistica for Windows 10.0 software.

## 3. Results

**Figure 4:**
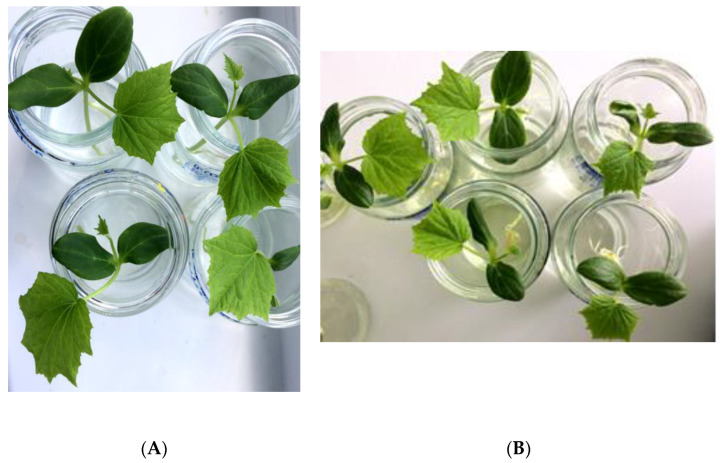
Cucumber seedlings after 14 days of cultivation. (**A**) Knop medium, (**B**) *Chlorella vulgaris* suspension.

The cucumber plants were grown on a nutrient medium with and without *Chlorella* suspension and did not differ from each other in appearance (Figure 4). Seedlings in both cases had a bright green color, with well-developed cotyledon leaves. The first true leaves in the experimental and control variants appeared on day 8, while the second real leaves began to form on day 12. The root system of the cucumbers by the fourteenth day of the experiment was well-developed in the experimental and control plants.

**Figure 5:**
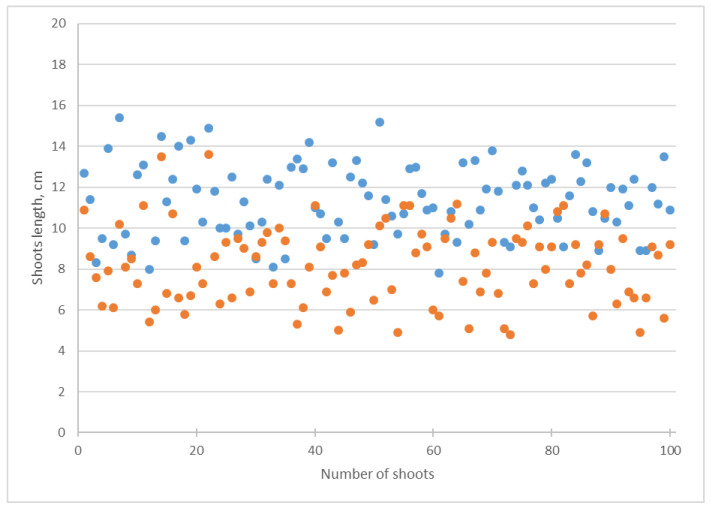
Influence of *Chlorella vulgaris* suspension on *Cucumis sativus* shoots length. Blue dots correspond to attribute values in the Knop solution; orange dots mean attribute values in *Chlorella vulgaris* suspension.

**Figure 6:**
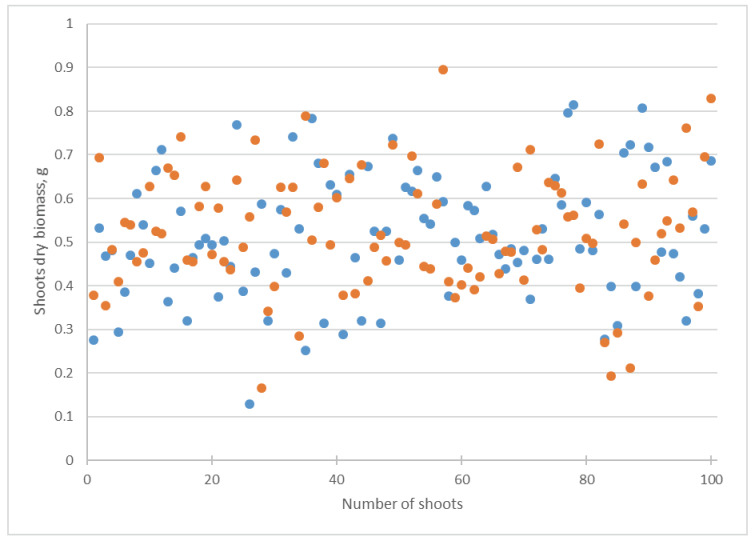
Influence of *Chlorella vulgaris* suspension on *Cucumis sativus* roots length. Blue dots correspond to attribute values in the Knop solution; orange dots mean attribute values in *Chlorella vulgaris* suspension.

During the cultivation on a Knop medium with the addition of *Chlorella* suspension, the length of the shoots was shortened (Figure 5). This change was confirmed by a decrease in the arithmetic mean and median from 11.30 to 8.15 cm (Table 1). The standard deviation of the shoots’ length increased from 1.77 to 1.92, while for the roots’ length, it decreased from 3.80 to 3.44 (Table 1). Alga suspension caused a decrease in the roots’ length arithmetic mean from 16.41 to 10.59 cm and a reduction in the median from 16.05 to 10.35 cm (Table 1, Figure 6).

*Chlorella* suspension affected the increase in the coefficient of variation in the cucumber shoots’ length from 15.66% cm to 23.44% cm and of the roots’ length from 23.09% cm to 32.51% cm (Table 1). The decrease in the length of the shoots and roots was statistically significant according to Student’s *t*-test (Table 1).

**Figure 7:**
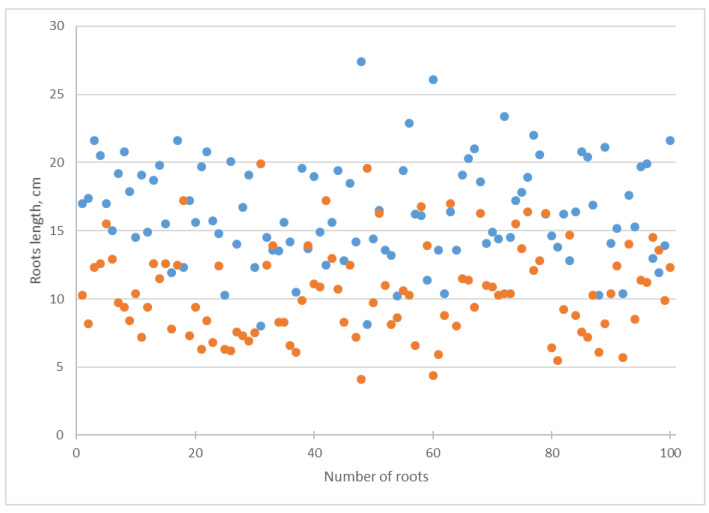
Influence of *Chlorella vulgaris* suspension on *Cucumis sativus* shoots dry biomass. Blue dots correspond to attribute values in the Knop solution; orange dots mean attribute values in *Chlorella vulgaris* suspension.

In the experiment with *Chlorella* suspension, the arithmetic mean of the shoots’ dry biomass increased from 0.50 to 0.53 g (Table 1, Figure 7), but this change was not reliable according to Student’s *t*-test (Table 1). The insignificance of these alterations was confirmed by other statistical indicators. The standard deviation and median did not change. The coefficient of variation increased insignificantly from 21.00 to 21.72 (Table 1).

**Figure 8:**
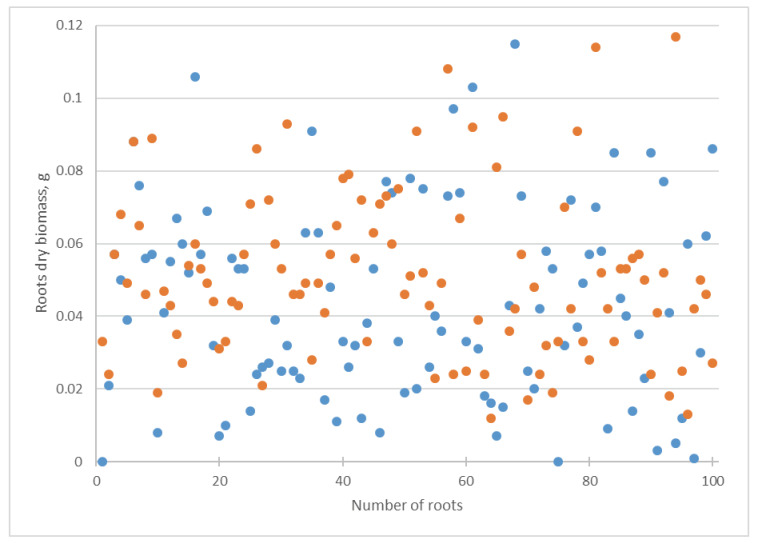
Influence of *Chlorella vulgaris* suspension on *Cucumis sativus* root dry biomass. Blue dots correspond to attribute values in the Knop solution; orange dots mean attribute values in *Chlorella vulgaris* suspension.

After using the *Chlorella* suspension, a significant increase in the arithmetic mean of the cucumber roots’ biomass from 0.04 to 0.05 g according to Student’s *t*-test was observed (Figure 8, Table 1). The increase in the median coincided with a change in the arithmetic mean. The values of the standard deviation and coefficient of variation decreased from 0.03 to 0.02 and from 59.60% to 37.02%, respectively (Table 1).

An increase in the cucumber shoots and roots biomass, together with a reduction in their length, is likely due to an increase in their thickness. It is likely that the suspension of the studied chlorella strain can activate the process of cucumber lateral growth, especially in the root zone.

It is known that the respiration rate of the roots in crops positively and linearly correlates with the level of dissolved O_2_ in the nutrient solution [57]. Thus, high levels of dissolved oxygen in nutrient solutions of an eco-hydroponic crop (agricultural crop + algae + hydroponic solution) were crucial for the respiration and root growth of crops and led to high yields and productivity [14].

It is likely that, in the hydroponic system with cucumber seedlings and *Chlorella* suspension, an increase in the root biomass was observed due to a high content of dissolved O_2_.

## 4. Discussion

Our investigation revealed that the addition of a suspension of *Chlorella vulgaris* into the Knop media caused a decrease in the cucumber shoot and root length, together with an increase in their dry biomass (Table 1, Figure 5, Figure 6, Figure 7 and Figure 8). Using several statistical indicators made it possible to estimate its influence more precisely. For example, the changes in the length and dry biomass of the shoots and roots were confirmed not only by the differences in the arithmetic mean but also by the median (Table 1). In the variants of the experiment with *Chlorella* suspension, a mostly increasing coefficient of variation was observed (Table 1). Only in the experiment regarding the roots’ dry biomass was a decrease in the coefficient of variation established.

It is necessary to note that roots are a basic plant organ that takes part in the consumption and transportation of water and nutrients, in the synthesis of biologically active substances (hormones, organic, and amino acids), and in fixing plants to the substrate [58,59]. Root biomass is one of the most important aspects of root functioning [60,61,62,63]. The dimensional and morphological characteristics of roots influence the size and development of the shoot and, therefore, future yields [58,64,65].

The grain yield of upland rice was raised in a quadratic fashion with an increase in the root length and roots’ dry weight. It was found that the roots’ dry weight was a better prognosticator of yield than the roots’ length or the shoots’ dry weight. Similar results were obtained for tropical legume cover crops [66,67].

Our research demonstrates that, under the influence of *Chlorella vulgaris* suspension, changes in the length of the shoots and roots do not always coincide with a difference in dry biomass. The change in biomass more accurately reflects the influence of algae since the shortening of shoots and roots was accompanied by an increase in their thickness.

It should be noted that reducing the length of the shoots and roots facilitates their further cultivation since shorter plants are less damaged if they are moved to other containers in the case of production necessity.

In our study, the positive influence of *Chlorella vulgaris* on cucumber roots’ dry biomass was detected. Very similar results were obtained in experiments with *Chlorella vulgaris* and *Mentha* spp. (mint) seedlings [68]. The maximal weight rise of the mint by 0.47 g was detected in the microalgae-containing and aerated variant of the experiment, while a minimal weight increase of 0.22 g was observed in the microalgae-free and non-aerated variant. The stimulating effect of *Chlorella sorokiniana* on maize roots was observed [69]. *C. sorokiniana* specifically increased the number of secondary roots. The promoting effect of *Chlorella* on the cucumber and tomato root and shoot growth was revealed during a study of this alga on the germination of the seeds of these plants [70]. The positive influence of algae on tomato roots has been detected in other studies [71]. During the co-cultivation of a tomato plant with algal inoculum, positive interactions between the microalgae and plant were detected. In these experiments, an increase in dissolved oxygen, together with effective root respiration, was observed. It is necessary to note that a highly developed root system supplies metabolic properties that activate nutrient uptake and accretion [71].

The growth of the cucumber root’s dry biomass after the influence of *Chlorella vulgaris* could be explained by the release of phytohormones or other biologically active substances produced by this alga. It is known that *Chlorella vulgaris* cells contain different amino acids, lipids, carbohydrates, pigments, and vitamins [35]. *Chlorella* cells are rich in macro- (Na, K, Ca, Mg, P) and microelements (Cr, Cu, Zn, Mn, Se, Fe), which are necessary for the functioning of plants [35]. This species demonstrated simulative effects on the expression of root traits and genes when connected with nutrient accession in sugar beet [72]. Representatives of the genus *Chlorella* (*Chlorella pyrenoidosa* and *Chlorella minutissima*) secreted auxin [17,19,20,21], which influenced root growth and development [73]. It was mentioned before that the algae stimulation mechanism is not clear and could be associated with the influence of several secondary metabolites [74]. Moreover, *Chlorella vulgaris* produces metabolites with antibiotic activity, which suppresses the growth of pathogenic microorganisms [74,75].

The positive effect of *Chlorella* species on growing plants in hydroponic systems has been discussed in previous investigations. The simulative effect of *Chlorella infusionum* on the development of tomato roots, root dry biomass, and root respiration rate was detected in a simple eco-hydroponic system [14]. The eco-hydroponic system consisted of a transparent container, algae-inoculated culture media, and materials for crop fixation.

Earlier, it was suggested that the influence of algae on hydroponic systems depends on the algal community (species), their density, growing plant, and climatic peculiarities [76]. Representatives of *Chlorella* mainly had a stimulating effect on these plants. As noted above, the positive effect of *Chlorella* suspension in hydroponics was discovered for a number of crops: tomato, maize, mint, Swiss chard, lettuce, pakchoi, rocket, spinach, and basil. This information, together with the results of our research, makes it possible to recommend algae of the genus *Chlorella* for wider use in the cultivation of agricultural plants on hydroponics. Moreover, the use of *Chlorella* not only saved on mineral nutrients but was provided an environmentally friendly approach.

The obtained results expand our knowledge of the authentic strain of *Chlorella vulgaris*, which is a kind of standard not only for the genus *Chlorella* but also for green algae in general. For a long time, strains of *Chlorella* have been used as model organisms in studies of plant physiology and biochemistry [77]. This strain was used in diverse biotechnological studies: the growth temperature range and fatty acid composition [78], the kinetics of growth and lipids accumulation during batch heterotrophic cultivation [79], the growth of the strains and associated bacteria in photobioreactors [80], the production of oligomannosidic glycans [81], and bio-compatible flotation [82].

However, the data regarding the use of authentic strains of *Chlorella vulgaris* for stimulating plant growth are very limited. This strain was applied to promote germination energy, germination, number, and the timing of ovaries, flowers, and fruits of *Capsicum annuum* L. (Bulgarian pepper) [83]. *Chlorella* suspension at a concentration of 2 *×* 10^6^ cells/mL increased the seed germination energy by 12%. The yield of peppers in the experimental group when adding *Chlorella* suspension was higher by 44% than the yield of the control group without suspension application. At the end of the growing season, the control plants exhibited signs of disease. Peppers treated with the suspension remained healthy, which indicated the strengthening of plant immunity.

The results of our study demonstrate that the authentic strain of *Chlorella vulgaris* has great potential for use in agriculture. New data regarding the possibility of a reference strain of *Chlorella vulgaris* to increase the dry biomass of cucumber root could make it possible for use as a growth promoter separately or as part of complex biological products. In addition, this strain is stored in many algae collections [84] and can be used by a wide range of researchers and the business community.

Thus, this study examining the influence of the authentic strain of *Chlorella vulgaris* on cucumber seedlings in a hydroponic system revealed the stimulative effect of alga on the roots’ biomass, which allows us to recommend it as a biostimulator for growth in hydroponic systems and for wider use in agriculture.

## Figures and Tables

**Table 1:** The effect of *Chlorella vulgaris* suspension on the length of shoots and roots of cucumbers.

Variant of Experiment	X_min_	X_max_	X ± S	σ	Me	CV, %	t
Shoots length, cm							
Knop medium	8.00	15.20	11.30 ± 0.18	1.77	11.30	15.66	-
*Chlorella* suspension	4.80	13.50	8.15 ± 0.19	1.92	8.15	23.44	12.07 *
Roots length, cm							
Knop medium	8.00	27.10	16.41 ± 0.38	3.80	16.05	23.09	-
*Chlorella* suspension	4.40	19.90	10.59 ± 0.34	3.44	10.35	32.51	11.38 *
Shoots dry biomass, g							
Knop medium	0.28	0.77	0.50 ± 0.01	0.11	0.49	21.00	-
*Chlorella* suspension	0.36	0.90	0.53 ± 0.01	0.11	0.49	21.72	1.48
Roots dry biomass, g							
Knop medium	0.001	0.12	0.04 ± 0.003	0.03	0.04	59.60	-
*Chlorella* suspension	0.02	0.11	0.05 ± 0.002	0.02	0.05	37.02	2.31 *

Notes. The number of measurements was 100. X_min_—minimum value of the attribute; X_max_—the maximum value of the attribute; X ± S—arithmetic mean and its error; Me—median; σ—standard deviation; CV—coefficient of variation; t—the values of Student’s coefficient. * marks the reliable values of Student’s criterion at *p* = 0.05.

## Data Availability

Not applicable.

## References

[1] Marfá O. (2000). Los cultivos sin suelo desde una perspectiva mediterránea. Recirculación en Cultivos Sin Suelo.

[2] Sowmya R.S., Warke V.G., Mahajan G.B., Raut M.R., Annapure U.S. (2022). Hydroponics: An Intensified Agriculture Practice to Improve Food Production. Rev. Agric. Sci..

[3] Ellis N.K., Jensen M., Larsen J., Oebker N. (1974). Nutriculture Systems—Growing Plants Without Soil. Station Bulletin-Dept. of Agricultural Economics.

[4] Sardare M.D., Admane S.V. (2013). A review on plant without soil. Int. J. Res. Eng. Technol..

[5] Beibel J.P. (1960). Hydroponics; The Science of Growing Crops Without Soil.

[6] Butler J.D., Oebker N.F. (2006). Hydroponics as a Hobby—Growing Plants without Soil.

[7] Macwan J., Pandya D., Pandya H. (2020). Review on soilless method of cultivation: Hydroponics. Int. J. Recent Sci. Res..

[8] Orsini F., Kahane R., Nono-Womdim R., Gianquinto G. (2013). Urban agriculture in the developing world: A review. Agron. Sustain. Dev..

[9] Khan S., Purohit A., Vadsaria N. (2020). Hydroponics: Current and future state of the art in farming. J. Plant Nutr..

[10] Hershey D.R. (1994). Solution Culture Hydroponics: History & Inexpensive Equipment. Am. Biol. Teach..

[11] Baudoin W.O., Winsor G.W., Schwarz M. (1990). Soilless Culture for Horticultural Crop Production.

[12] Araghian S., Bagherzadeh A., Sadrabadi R. (2015). Effect of brown algae and vermicompost application on some cherry tomato traits in hydroponic system. Agroecol. J..

[13] Schwarz D., Krienitz L. (2004). Do Algae Cause Growth-Promoting Effects on Vegetables and Growth Hydroponically.

[14] Zhang J., Wang X., Zhou Q. (2016). Co-cultivation of *Chlorella* spp and tomato in a hydroponic system. Biomass Bioenergy.

[15] Ördög V. Beneficial effects of microalgae and cyanobacteria in plant/soil-systems, with special regard to their auxin- and cytokinin-like activity. Proceedings of the International Workshop and Training Course on Microalgal Biology and Biotechnology.

[16] Van Staden J. Occurrence and Potential Physiological Effects of Algal Plant Growth Regulators. Proceedings of the International Workshop and Training Course on Microalgal Biology and Biotechnology.

[17] Mazur H., Konop A., Synak R. (2001). Indole-3-acetic acid in the culture medium of two axenic green microalgae. J. Appl. Phycol..

[18] Tarakhovskaya E.R., Maslov Y.I., Shishova M.F. (2007). Phytohormones in algae. Russ. J. Plant Physiol..

[19] Stirk W., Bálint P., Tarkowská D., Novák O., Strnad M., Ördög V., van Staden J. (2013). Hormone profiles in microalgae: Gibberellins and brassinosteroids. Plant Physiol. Biochem..

[20] Stirk W.A., Ördög V., Novák O., Rolèík J., Strnad M., Bálint P., Staden J. (2013). Auxin and cytokinin relationships in 24 microalgal strains. J. Phycol..

[21] Lu Y., Xu J. (2015). Phytohormones in microalgae: A new opportunity for microalgal biotechnology?. Trends Plant Sci..

[22] Kapoore R.V., Wood E.E., Llewellyn C.A. (2021). Algae biostimulants: A critical look at microalgal biostimulants for sustainable agricultural practices. Biotechnol. Adv..

[23] Addy M.M., Kabir F., Zhang R., Lu Q., Deng X., Current D., Griffith R., Ma Y., Zhou W., Chen P. (2017). Co-cultivation of microalgae in aquaponic systems. Bioresour. Technol..

[24] Borowitzka M.A. (1995). Microalgae as sources of pharmaceutical and other biologically active compounds. J. Appl. Phycol..

[25] Ravina I., Paz E., Sofer Z., Marm A., Schischa A., Sagi G., Yechialy Z., Lev Y. (1997). Control of clogging in drip irrigation with stored treated municipal sewage effluent. Agric. Water Manag..

[26] Huizebos E.M., Adema D.M.M., Dirven-van Breemen E.M., Henzen L., van Dis W.A., Herbold H.A., Hoekstra J.A., Baerselman R., van Gestel C.A.M. (1993). Phytotoxicity studies with *Latuca sativa* in soil and nutrient solution. Environ. Toxicol. Chem..

[27] Gonzales L.E., Canizares R.O., Baena S. (1997). Efficiency of ammonia and phosphorus removal from a Colombian agroindustrial wastewater by the microalgae *Chlorealla vulgaris* and *Scenedesmus*. Bioresour. Technol..

[28] Yamaguchi K. (1996). Recent advances in microalgal bioscience in Japan, with special reference to utilization of biomass and metabolites: A review. J. Appl. Phycol..

[29] Lee K., Lee C.-G. (2001). Effect of light/dark cycles on wastewater treatments by microalgae. Biotechnol. Bioprocess Eng..

[30] Jeon J.K. (2006). Effect of *Chlorella* addition on the quality of processed cheese. J. Korean Soc. Food. Sci. Nutr..

[31] Spolaore P., Joannis-Cassan C., Duran E., Isamber A. (2006). Commercial applications of microalgae. J. Biosci. Bioeng..

[32] Barrow C., Shahidi F. (2007). Marine Nutraceuticals and Functional Foods.

[33] Sheih I.-C., Fang T.J., Wu T.-K., Lin P.-H. (2009). Anticancer and Antioxidant Activities of the Peptide Fraction from Algae Protein Waste. J. Agric. Food Chem..

[34] Beheshtipour H., Mortazavian A.M., Mohammadi R., Sohrabvandi S., Khosravi-Darani K. (2013). Supplementation of *Spirulina platensis* and *Chlorella vulgaris* Algae into Probiotic Fermented Milks. Compr. Rev. Food Sci. Food Saf..

[35] Safi C., Zebib B., Merah O., Pontalier P.-Y., Vaca-Garcia C. (2014). Morphology, composition, production, processing and applications of *Chlorella vulgaris*: A review. Renew. Sustain. Energy Rev..

[36] Coronado-Reyes J.A., Salazar-Torres J.A., Juárez-Campos B., González-Hernández J.C. (2022). *Chlorella vulgaris*, a microalgae important to be used in Biotechnology: A review. Food Sci. Technol..

[37] Leng S., Jiao H., Liu T., Pan W., Chen J., Chen J., Huang H., Peng H., Wu Z., Leng L. (2022). Co-liquefaction of *Chlorella* and soybean straw for production of bio-crude: Effects of reusing aqueous phase as the reaction medium. Sci. Total Environ..

[38] Gitau M.M., Farkas A., Balla B., Ördög V., Futó Z., Maróti G. (2021). Strain-Specific Biostimulant Effects of *Chlorella* and *Chlamydomonas* Green Microalgae on *Medicago truncatula*. Plants.

[39] Bertoldi F.C., Sant’Anna E., Barcelos-Oliveira J.L. (2009). *Chlorella Vulgaris* Cultivated in Hydroponic Wastewater. Acta Hortic..

[40] Yousif Y.I.D., Mohamed E.S., El-Gendy A.S. (2022). Using chlorella vulgaris for nutrient removal from hydroponic wastewater: Experimental investigation and economic assessment. Water Sci. Technol..

[41] Žunić V., Jafari T.H., Grabić J., Đurić S., Stamenov D. (2022). Hydroponic systems: Exploring the balance between co-cultivation of *Chlorella vulgaris* and Swiss chard (*Beta vulgaris* L. subsp. cicla). J. Appl. Phycol..

[42] Dasgan H.Y., Kacmaz S., Arpaci B.B., Ikiz B., Gruda N.S. (2023). Biofertilizers Improve the Leaf Quality of Hydroponically Grown Baby Spinach (*Spinacia oleracea* L.). Agronomy.

[43] Fimbres-Acedo Y.E., Servín-Villegas R., Garza-Torres R., Endo M., Fitzsimmons K.M., Emerenciano M.G.C., Magal-lón-Servín P., López-Vela M., Magallón-Barajas F.J. (2020). Hydroponic horticulture using residual waters from *Oreochromis niloticus* aquaculture with biofloc technology in photoautotrophic conditions with *Chlorella microalgae*. Aquacult. Res..

[44] Ergun O., Dasgan H., Isık O. (2020). Effects of microalgae *Chlorella vulgaris* on hydroponically grown lettuce. Acta Hortic..

[45] Mallick P.K. (2022). Evaluating Potential Importance of Cucumber (*Cucumis sativus* L.—Cucurbitaceae): A Brief Review. Int. J. Appl. Sci. Biotechnol..

[46] Mikherjee P.K., Neema N.K., Maity N., Sarkar B.K. (2013). Phytochemical and Therapeutic Potential of Cucumber.

[47] Tatlioglu T., Kalloo G., Bergh B.O. (1993). 13—Cucumber: *Cucumis sativus* L.. Genetic Improvement of Vegetable Crops.

[48] Sharma V., Sharma L., Sandhu K.S., Nayik G.S., Gull A. (2020). Cucumber (*Cucumis sativus* L.). Antioxidants in Vegetables and Nuts—Properties and Health Benefits.

[49] Wilcox G.L., Offer U.S., Omojola J.T. (2016). Profitability of Cucumber (*Cucumis sativa* L.) Production in local Government Area of River State, Nigeria. J. Adv. Stud. Agric. Biol. Environ. Sci..

[50] Chandra S., Khan S., Avula B., Lata H., Yang M.H., ElSohly M.A., Khan I.A. (2014). Assessment of Total Phenolic and Flavonoid Content, Antioxidant Properties, and Yield of Aeroponically and Conventionally Grown Leafy Vegetables and Fruit Crops: A Comparative Study. Evid.-Based Complement. Altern. Med..

[51] Beijerinck M.W. (1890). Culturversuche mit Zoochlorellen, Lichenengonidien und anderen niederen Algen. Ztg. Bot..

[52] Guiry M.D., Guiry G.M., AlgaeBase (2022). World-Wide Electronic Publication, National University of Ireland, Galway. https://www.algaebase.org.

[53] Bischoff H.W., Bold H.C. (1963). Phycological Stydies IV. Some Soil Algae from Enchanted Rock and Related Algal Species.

[54] Bold H.C. (1942). The cultivation of algae. Bot. Rev..

[55] Abdulganieva D.I., Bombina L.K., Nazarova M.D., Khalfina T.N. (2016). On the occasion of the 140th anniversary of the birth of the Professor N.K. Goryaev. Russ. J. Hematol. Transfusiol..

[56] Webster R. (2001). Statistics to support soil research and their presentation. Eur. J. Soil Sci..

[57] Zheng Y., Wang L., Dixon M. (2007). An upper limit for elevated root zone dissolved oxygen concentration for tomato. Sci. Hortic..

[58] Mishra P., Singh U., Pandey C.M., Mishra P., Pandey G. (2019). Application of student’s *t*-test, analysis of variance, and co-variance. Ann. Card. Anaesth..

[59] Leskovar D.I., Stofella P.J. (1995). Vegetable Seedling Root Systems: Morphology, Development, and Importance. HortScience.

[60] Yang C., Yang L., Yang Y., Ouyang Z. (2004). Rice root growth and nutrient uptake as influenced by organic manure in continuously and alternately flooded paddy soils. Agric. Water Manag..

[61] Samejima H., Kondo M., Ito O., Nozoe T., Shinano T., Osaki M. (2005). Characterization of root systems with respect to morphological traits and nitrogen-absorbing ability in new plant type of tropical rice lines. J. Plant Nutr..

[62] Wang H., Inukai Y., Yamauchi A. (2006). Root development and nutrient uptake. Crit. Rev. Plant Sci..

[63] Yang L.X., Wang Y.L., Kobayashi K., Zhu J.G., Huang J.Y., Yang H.J., Wang Y.X., Dong G.C., Liu G., Han Y. (2008). Seasonal changes in the effects of free-air CO_2_ enrichment (FACE) on growth, morphology and physiology of rice root at three levels of nitrogen fertilization. Glob. Chang. Biol..

[64] Yang J., Zhang H., Zhang J. (2012). Root Morphology and Physiology in Relation to the Yield Formation of Rice. J. Integr. Agric..

[65] Barber S.A., Silberbush M., Barber S.A., Bouldin D.R. (1984). Plant root morphology and nutrient uptake. Roots, Nutrient and Water Influx, and Plant Growth.

[66] Fageria N.K., Moreira A. (2011). The role of mineral nutrition on root growth of crop plants. Advances in Agronomy.

[67] Gregory P.J., Peterson G.A. (1994). Root growth and activity. Physiology and Determination of Crop Yield.

[68] Uyar G.E., Mısmıl N. (2022). Symbiotic association of microalgae and plants in a deep water culture system. PeerJ.

[69] Martini F., Beghini G., Zanin L., Varanini Z., Zamboni A., Ballottari M. (2021). The potential use of *Chlamydomonas reinhardtii* and *Chlorella sorokiniana* as biostimulants on maize plants. Algal Res..

[70] Bumandalai O. (2019). Effect of *Chlorella vulgaris* as a biofertilizer on germination of tomato and cucumber seeds. Int. J. Aquat. Biol..

[71] Supraja K.V., Behera B., Balasubramanian P. (2020). Performance evaluation of hydroponic system for co-cultivation of microalgae and tomato plant. J. Clean. Prod..

[72] Barone V., Baglieri A., Stevanato P., Broccanello C., Bertoldo G., Bertaggia M., Cagnin M., Pizzeghello D., Moliterni V.M.C., Mandolino G. (2017). Root morphological and molecular responses induced by microalgae extracts in sugar beet (*Beta vulgaris* L.). J. Appl. Phycol..

[73] Woodward A.W., Bartel B. (2005). Auxin: Regulation, action, and interaction. Ann. Bot..

[74] Dvoretsky D., Dvoretsky S., Temnov M., Markin I., Akulinin E., Golubyatnikov O., Ustinskaya Y., Eskova M. (2019). Experimental research into the antibiotic properties of *Chlorella vulgaris* algal exometabolites. Chem. Eng. Trans..

[75] Almalki M.A., Khalifa A.Y., Alkhamis Y.A. (2022). In vitro Antibiosis of *Chlorella vulgaris* Extract against the Phytopathogen, *Stenotrophomonas maltophilia*. J. Pure Appl. Microbiol..

[76] Schwarz D., Gross W. (2004). Algae affecting lettuce growth in hydroponic systems. J. Hortic. Sci. Biotechnol..

[77] Burja A.M., Tamagnini P., Bustard M.T., Wright P.C. (2001). Identification of the green alga, *Chlorella vulgaris* (SDC1), using cyanobacteria-derived 16S rDNA primers: Targeting the chloroplast. FEMS Microbiol. Lett..

[78] Xu J., Hu H. (2013). Screening high oleaginous *Chlorella* strains from different climate zones. Bioresour. Technol..

[79] Sakarika M., Kornaros M. (2017). Kinetics of growth and lipids accumulation in *Chlorella vulgaris* during batch heterotrophic cultivation: Effect of different nutrient limitation strategies. Bioresour. Technol..

[80] Lakaniemi A.-M., Intihar V.M., Tuovinen O.H., Puhakka J.A. (2011). Growth of *Chlorella vulgaris* and associated bacteria in photobioreactors. Microb. Biotechnol..

[81] Mócsai R., Figl R., Troschl C., Strasser R., Svehla E., Windwarder M., Thader A., Altmann F. (2019). N-glycans of the microalga *Chlorella vulgaris* are of the oligomannosidic type but highly methylated. Sci. Rep..

[82] Matho C., Schwarzenberger K., Eckert K., Keshavarzi B., Walther T., Steingroewer J., Krujatz F. (2019). Bio-compatible flotation of *Chlorella vulgaris*: Study of zeta potential and flotation efficiency. Algal Res..

[83] Oleinikova D.V., Sukhanova N.V. The use of *Chlorella vulgaris* Beijer. suspension as a growth stimulator of greenhouse crops. Modern aspects of the study of plant ecology. Proceedings of the VII International Youth Competition-Conference, M. Akmullah Bashkir State Pedagogical University.

[84] Müller J., Friedl T., Hepperle D., Lorenz M., Day J.G. (2005). Distinction between multiple isolates of *Chlorella vulgaris* (Chlorophyta, Trebouxiophyceae) and testing for conspecificity using amplified fragment length polymorphism and its rDNA sequences. J. Phycol..

